# Relationship between grief and coping strategies among nurses dealing with patient deaths: a descriptive, cross-sectional, correlational study

**DOI:** 10.1186/s12904-025-01790-7

**Published:** 2025-05-26

**Authors:** Loujain Sharif, Khalid Almutairi, Ibrahim Alnasser, Zalikha Attar, Alaa Mahsoon, Aisha Alhofaian, Budour Almutairi, Yaser Alqahtani, Afnan Tunsi, Sara Yaghmour, Fayez Bokhari, Rebecca Wright

**Affiliations:** 1 Psychiatric and Mental Health Nursing, Faculty of Nursing, King Abdulaziz University, Jeddah, 21551 KSA Saudi Arabia; 2 King Fahad Armed Forces Hospital (KFAFH), Jeddah, KSA Saudi Arabia; 3 Medical Surgical nursing, Faculty of Nursing, King Abdulaziz University, Jeddah, KSA Saudi Arabia; 4 Johns Hopkins University School of Nursing, Baltimore, MD USA

**Keywords:** Grief, Coping, Nursing, Patient death, Saudi Arabia, Cross-sectional study, Online survey

## Abstract

**Background:**

Nurses often face significant emotional distress and grief when dealing with patient deaths, especially in acute hospital settings. Despite extensive literature offering guidance on nursing practices for providing optimal care to terminally ill patients and their grieving families, there is a scarcity of empirical research examining nurses’ experiences of grief following patient deaths in Middle Eastern contexts. This study aimed to assess the relationship between grief and coping strategies among nurses experiencing patient death in an acute hospital in Saudi Arabia.

**Methods:**

A cross-sectional study was conducted using an online survey distributed to nurses at King Fahad Armed Forces Hospital in Jeddah. Data from 382 nurses were analyzed using SPSS software. Descriptive statistics, bivariate analyses, Pearson’s chi-square test, Student’s t-test, one-way ANOVA, and regression analysis were employed to examine the associations between grief levels, coping strategies, and various socio-demographic and professional characteristics.

**Results:**

Among the participants, 80% were aged 25–40 years, and 50% were married. Most nurses (85.4%) reported normal levels of grief. Coping strategies’ mean scores ranged from 2.60 to 5.52. Grief levels showed significant correlations with nationality, received support, and intervention type. Nurses with high or severe grief levels had significantly higher mean scores for problem-focused, emotion-focused, and avoidant coping strategies (*p* < 0.001). A significant positive linear correlation was found between total coping scores and total grief scores. Regression analysis indicated that 21.3% of the variance in total coping scores was explained by total grief scores.

**Conclusions:**

The study highlights that while nurses employ personal coping strategies, there is a need for additional, culturally tailored support to manage grief effectively. Implementing structured support systems may enhance nurses’ coping mechanisms and overall well-being when facing patient deaths.

## Background

In addition to nurses’ responsibilities of providing physical care for end-of-life patients, they also provide care for emotional distress, enduring grief, and anxiety experienced by patients and care partners in anticipation of impending loss [1]. Nursing practice is driven by compassion, and ongoing exposure to patients’ suffering and loss, combined with high work demands, can lead to an overwhelming sense of grief among nurses [2].

Grief is a complex reaction to loss, encompassing not only the expected emotional response but also the physical, cognitive, behavioral, social, and philosophical dimensions [3]. The nature of grief cannot be confined to a single domain of origin, as grief experiences can impact individuals at psychological, sociological, and physiological levels, either individually or concurrently [3]. Factors influencing how healthcare professionals respond to patient death are inherently personal, and it has been suggested that life experiences and encounters with mortality shape the ways in which these responses are expressed and which coping strategies are used [4, 5].

Many hospital nurses, despite their expertise in advanced medical treatments and patient care, often find themselves ill-prepared to attend to patients in the final stages of life [6]. When nurses experience grief at the death of a patient in their care, they report feeling powerlessness, guilt, fear, denial, shock, anger, crying, sadness, regret, compassion, relief, helplessness, and fear of the family’s reactions [7–12]. Certain factors have been found to exacerbate the incidence of grief among nurses during patient death. These include deaths of a younger person [10, 11], duration of death (sudden/unexpected or drawn out), relationship with the patient or family, and if the patient dies without family [10, 11].

As grief is not solely an emotional process (it manifests in physical, cognitive, and psychological aspects of health), effective coping strategies are essential for resilience to the negative implications of grief. Coping involves successful stressor management. It assesses strategies employed by individuals to manage stress [13]. Coping strategies are methods employed by nurses to manage the demanding situations they encounter [14, 15]. Coping strategies can be classified as adaptive or maladaptive [16]. Adaptive coping strategies focus on addressing both problems and emotions associated with a situation, whereas maladaptive strategies revolve around avoidance [16]. Effective coping results in more positive long-term mental health outcomes and contributes to enhanced overall well-being after a significant life event [17]. Conversely, a lack of successful coping strategies is linked to a higher occurrence of persistent mental health problems, such as anxiety, depression, and post-traumatic stress disorder [18]. For nurses, various work factors can influence coping either negatively or positively, such as workload, staffing, and previous experience with death [10, 11].

Different coping strategies successfully used by nurses in response to grief over the loss of a patient include talking about the death in the workplace [10] and strong nurse-to-nurse relationships with supportive listening, moral support, and informal discussions [8, 12]. Formal support is an effective method for coping, including debriefing, clinical supervision, and counseling services [12]. Other nurse-reported coping mechanisms include relaxation techniques [10], solitary reflections, socializing, family time, exercise, and leisure pursuits [12]. In addition, beliefs about death can aid coping, normalizing it as life’s natural course, with prayer and acceptance of death as divinely ordained, providing solace in some instances [12]. Some nurses find that the act of providing nursing care and responding to others needs was in and of itself, a method of coping, for example, satisfaction in helping families grieve or ensuring successful organ donation after a patient’s death [12].

Despite a rich body of literature guiding nursing practices in providing optimal care for terminally ill patients and their grieving families [19, 20], there is limited empirical research exploring nurses’ experiences of grief after patient death in Middle Eastern contexts. The cultural contexts nurses work in is particularly important to understand as professional and personal practices and preferences will be influenced by the norms of that culture. For example, there are specific spiritual practices in Arab and Muslim cultures such as positioning a dying person towards Mecca, and belief in the power of God to heal at any stage, the latter of which has significant implications for timing and presence goals of care conversations [21]. How death and loss is subsequently understood and processed will be directly influenced by these cultural norms and as such, interventions and support must take these contexts into consideration if they are to be effective [22]. This study sought to assess the relationship between grief and coping strategies among nurses dealing with patient deaths in an acute hospital setting in Saudi Arabia.

## Methods

### Design

We employed a descriptive, cross-sectional, correlational research design.

### Setting and participants

We recruited all nursing staff members who had experienced and cared for one or more patients who died at King Fahad Armed Forces Hospital in Jeddah. Nurses were recruited from intensive care units, medical, nephrology, surgical pediatric, and VIP wards. The main inclusion criterion was nurses who had experienced the death of a patient. Based on power analysis, the sample size was estimated at 304 participants, determining n = (z)2 p (1 − p)/d2 and a 6% margin of error.

### Measures

We conducted a survey comprised of three sections. The first collected socio-demographic data included age, sex, nationality, marital status, years of experience, educational attainment, working unit, and number of patient deaths experienced during the previous year. The second section used the validated Grief Traits and State Scale for Nurses (GSSN) [23]. This 31-item self-report grieving measure is split into two dimensions. The first was a set of 12 items measuring grieving features (individual characteristics indicating the nurse’s current state of mind) scored on a 5-point Likert Scale. The second dimension also used a 5-point Likert Scale, which comprised 19 items that gauged nurses’ level of sadness. Present study testing of internal consistency found Cronbach’s alpha varied between 0.897 and 0.86 for all 31 items. The third section used the validated Brief Coping Orientation to Problems Experienced Inventory (Brief-COPE), with an overall Cronbach’s alpha range 0.912-0.935. This scale uses a 4-point Likert Scale to measure 28 items evaluating the ways in which individuals manage stressful life experiences, split into three subscales: problem-focused, emotion-focused, and avoidant coping. The wide definition of “coping” refers to the strategies used to lessen the distress brought on by unpleasant situations and lists a variety of coping strategies participants can select from, including planning, positive reframing, active coping, self-distraction, denial, substance abuse, behavioral disengagement, emotional support, humor, venting, acceptance, self-blame, and religion.

### Ethical considerations

Ethics approval was obtained from the King Fahad Armed Forces Hospital Ethical Research Committee (Ref. no. REC 638). The principles of the Declaration of Helsinki were followed [24]. Before enrolling in the survey, participants received an electronic informed consent form, including information about the study’s purpose, inclusion, and exclusion criteria, their ability to accept or reject participation, and the anonymous nature of the online survey.

### Data collection

The survey was sent to the nursing staff via their professional email addresses in December 2023 and remained available until February 2024. Data collection followed an approach that our team had previously successfully used, wherein senior nursing staff supported recruitment among clinical units [25].

### Data analysis

Data were analyzed using the IBM SPSS statistical software for Windows (version 26.0; IBM Corp., Armonk, NY, USA). Descriptive statistics (mean, standard deviation, frequency, and percentage) were used to ascertain the categorical variables. We used Pearson’s chi-square test and odds ratios to assess and measure the association between nurses’ categorical socio-demographic and professional characteristics and their grief levels. We used Student’s t-test for independent samples and one-way analysis of variance followed by a post hoc test (Tukey’s) to compare the mean values of quantitative variables (three factors of coping and total coping score) in relation to the demographic and professional characteristics of nurses, as well as the three levels of grief trait, grief state, and total grief. Pearson’s correlation was calculated to measure the relationship between the scores of the three factors, the total coping score, and the scores for grief trait, grief state, and total grief score. We conducted a simple linear regression analysis to quantify the linear relationship between the nurses’ total coping and grief scores. In this work, a p-value of ≤ 0.05 was considered statistically significant to ensure the precision of the results.

## Results

In total, 382 nurses participated, with approximately 80% aged between 25 and 40 years. Women accounted for 86.9% of participants. More than 50% of the sample were married, and 59.4% were Filipino nationals. More than 90% had a bachelor’s degree in nursing experience, and 61.8% had 1 to 10 years of nursing experience. These nurses’ working units were mostly intensive care units of different specialties (e.g., cardiac, pediatric, and neonatal) as well as emergency rooms, medical wards, surgical wards, Very Important Persons (VIP) wards, and pediatric wards. Among the 382 nurses, 342 (89.5%) experienced patient deaths during their working period, 62% had witnessed one to five patient deaths, and 14.6% had witnessed more than 10 deaths. Only 18.6% and 22.5% had received training and support, respectively, to deal with death incidents during their working period. The types of interventions these nurses engaged in included team debriefs (29.6%), psychological therapy (26.7%), and spiritual support (18.6%) (Table 1).

**Table 1:** Distribution of socio-demographic and professional characteristics of nurses

Characteristics	No. (%)
**Age groups**	
<=25	8 (2.3)
25–30	82 (24.0)
31–50	112 (32.7)
36–40	81 (23.7)
41–45	19 (5.6)
46–50	24 (7.0)
>=51	16 (4.7)
**Gender**	
Male	46 (13.5)
Female	296 (86.5)
**Marital status**	
Single	139 (40.6)
Married	188 (55.0)
Divorced/Widow	15 (4.4)
**Nationality**	
Saudi	45 (13.2)
Indian	79 (23.1)
Filipino	212 (62.0)
Other	6 (1.8)
**Level of nursing**	
Diploma	9 (2.6)
Bachelor	322 (94.2)
Master	11 (3.2)
**Nursing experience (in years)**	
< 1	46 (13.5)
5-Jan	111 (32.5)
10-Jun	102 (29.8)
15-Nov	55 (16.1)
> 15	28 (8.2)
**Working unit**	
Intensive Care unit	44 (12.9)
Cardiac intensive care unit	37 (10.8)
Pediatric cardiac Intensive care unit	14 (4.1)
Pediatric Intensive care unit	11 (3.2)
Neonatal Intensive care unit	44 (12.9)
Emergency room	49 (14.3)
Medical ward	58 (17.0)
Nephro ward	12 (3.5)
Surgical ward	45 (13.2)
VIP ward	9 (2.6)
Pediatric ward	17 (5.0)
Other	2 (0.6)
**How many patients died?**	
1 to 5	212 (62.0)
6 to 10	80 (23.4)
> 10	50 (14.6)
**Have received training**	
Yes	81 (23.7)
No	261 (76.3)
**Have received support**	
Yes	68 (19.9)
No	274 (80.1)
**Type of interventions**	
Bereavement Counseling	45 (13.2)
One-to-One Debriefs with Team Leader or Other Leadership	30 (8.8)
Psychological Therapy	90 (26.3)
Spiritual Support	63 (18.4)
Team Debriefs	107 (31.3)
Other	7 (2.0)

The level of grief trait, grief state, and total grief level among nurses facing patient death during their working period is given in (Table 2), where most nurses reported a normal level of grief trait (85.4%), grief state (85.7%), and total grief (86%). However, approximately 4% of the nurses reported severe grief.

**Table 2:** Levels of grief trait, grief State, and total grief among nurses

Grief and its components	No. (%)
**Grief Trait**	
Normal level	292 (85.4)
High level	35 (10.2)
Severe level	15 (4.4)
**Grief state**	
Normal level	293 (85.7)
High level	35 (10.2)
Severe level	14 (4.1)
**Total Grief**	
Normal level	294 (86.0)
High level	32 (9.4)
Severe level	16 (4.7)

The different components of coping mean values ranged from 2.60 to 5.52, with religion having the highest mean value. The emotion-focused coping factor had the highest mean value (24.54), followed by problem-focused coping (18.78) and avoidant coping (12.95). The mean coping score was 56.27 (Table 3).

**Table 3:** Descriptive statistics of different components, three factors and total scores of coping among nurses

Components of Coping instrument	Minimum	Maximum	Mean	Sd.
**Components**				
Self- distraction	2	8	4.54	1.67
Active Coping	2	8	5.12	1.79
Denial	2	8	3.04	1.31
Substance Use	2	8	2.60	0.96
Use of Emotional support	2	8	4.19	1.68
Use of Instrumental support	2	8	4.20	1.71
Behavioral Disengagement	2	8	3.12	1.42
Venting	2	8	3.67	1.40
Positive Reframing	2	8	5.01	1.88
Planning	2	8	4.46	1.74
Humor	2	8	2.64	1.13
Acceptance	2	8	5.52	1.76
Religion	2	8	5.61	1.84
**Factors**				
Self-blame	2	8	2.90	1.27
Problem Focused Coping	8	32	18.78	6.0
Emotion Focused Coping	12	48	24.54	6.13
Avoidant Coping	8	32	12.95	3.68
Total score	28	112	56.27	14.2

The association between nurses’ socio-demographic and professional characteristics and their grief levels showed a statistically significant association with nationality, received support, and intervention type. For nationality, a higher proportion (46.7%) of Saudi national nurses had a risk level of grief when facing patient deaths during their working hours, followed by 11.4% of Indian nurses and 8.5% of Filipino nurses, which showed a highly statistically significant difference (*p* < 0.0001). Only 5.9% of nurses who received support had an ‘at-risk’ level of grief, compared with 16.1% of nurses who did not receive support (*p* = 0.031). Nurses who underwent psychological therapy had a higher level of grief (24.4%), whereas 7.5% of those who underwent team debriefs had a higher level of grief (*p* = 0.018) (Table 4).

**Table 4:** Association between socio-demographic & professional characteristics of nurses and level of their grief

Characteristics	Grief Level		Χ^2^-value	*p*-value
	Normal	Risk		
**Age groups**				
<=25	6 (75.0)	2 (25.0)	12.46	0.052
25–30	63 (76.8)	19 (23.2)		
31–50	97 (86.6)	15 (13.4)		
36–40	71 (87.7)	10 (12.3)		
41–45	18 (94.7)	1 (5.3)		
46–50	23 (95.8)	1 (4.2)		
>=51	16 (100.0)	0 (0.0)		
**Gender**				
Male	39 (84.8)	7 (15.2)	0.06	0.804
Female	255 (86.1)	41 (13.9)		
**Marital status**				
Single	120 (86.3)	19 (13.7)	2.77	0.251
Married	159 (84.6)	29 (15.4)		
Divorced/Widow	15 (100.0)	0 (0.0)		
**Nationality**				
Saudi	24 (53.3)	21 (46.7)	46.55	< 0.0001
Indian	70 (88.6)	9 (11.4)		
Filipino	194 (91.5)	18 (8.5)		
Other	6 (100.0)	0 (0.0)		
**Level of nursing**				
Diploma	6 (66.7)	3 (33.3)	3.06	0.217
Bachelor	279 (86.6)	43 (13.4)		
Master	9 (81.8)	2 (18.2)		
**Nursing experience (in years)**				
< 1	38 (82.6)	8 (17.4)	5.96	0.202
5-Jan	92 (82.9)	19 (17.1)		
10-Jun	88 (86.3)	14 (13.7)		
15-Nov	48 (87.3)	7 (12.7)		
> 15	28 (100.0)	0 (0.0)		
**Working unit**				
Intensive Care unit	40 (90.9)	4 (9.1)	8.79	0.642
Cardiac intensive care unit	32 (86.5)	5 (13.5)		
Pediatric cardiac Intensive care unit	13 (92.9)	1 (7.1)		
Pediatric Intensive care unit	9 (81.8)	2 (18.2)		
Neonatal Intensive care unit	38 (86.4)	6 (13.6)		
Emergency room	42 (85.7)	7 (14.3)		
Medical ward	50 (86.2)	8 (13.8)		
Nephro ward	11 (91.7)	1 (8.3)		
Surgical ward	38 (84.4)	7 (15.6)		
VIP ward	8 (88.9)	1 (11.1)		
Pediatric ward	11 (64.7)	6 (35.3)		
Other	2 (100.0)	0		
**How many patients**				
1 to 5	181 (85.4)	31 (14.6)	0.8	0.671
6 to 10	68 (85.0)	12 (15.0)		
> 10	45 (90.0)	5 (10.0)		
**Have received training**				
Yes	70 (86.4)	11 (13.6)	0.02	0.893
No	224 (85.8)	37 (14.2)		
**Have received support**				
Yes	64 (94.1)	4 (5.9)	4.68	0.031
No	230 (83.9)	44 (16.1)		
**Type of interventions**				
Bereavement Counseling	40 (88.9)	5 (11.1)	13.62	0.018
One-to-One Debriefs with Team Leader or Other Leadership	25 (83.3)	5 (16.7)		
Psychological Therapy	68 (75.6)	22 (24.4)		
Spiritual Support	55 (87.3)	8 (12.7)		
Team Debriefs	99 (92.5)	8 (7.5)		
Other	7 (100.0)	0 (0.0)		

The comparison of the mean values of the three coping factors (problem-focused coping, emotion-focused coping, and avoidant coping) and the total coping score in relation to the nurses’ socio-demographic and professional characteristics showed a statistically significant difference in relation to nurses’ working units. The mean values of the three factors and the total scores were significantly higher among nurses working in the pediatric ward (Table 5).

**Table 5:** Mean values of three factors and total score of Cope instrument based on socio-demographic & professional characteristics

Characteristics	Problem Focused Coping		Emotion Focused Coping		Avoidant Coping		Coping total score	
	Mean (Sd.)	*p*-value	Mean (Sd.)	*p*-value	Mean (Sd.)	*p*-value	Mean (Sd.)	*p*-value
**Age groups**								
<=25	19.75 (3.9)	0.454	26.50 (3.5)	0.72	14.88 (3.0)	0.153	61.12 (5.6)	0.727
25–30	18.09 (5.6)		24.87 (6.4)		13.74 (3.8)		56.69 (14.4)	
31–50	19.17 (6.1)		24.35 (5.8)		12.47 (3.3)		55.99 (13.7)	
36–40	18.26 (6.2)		24.16 (6.5)		12.85 (4.2)		55.27 (15.5)	
41–45	20.89 (5.4)		26.21 (3.6)		12.63 (3.0)		59.73 (10.1)	
46–50	18.17 (7.0)		23.42 (6.9)		12.17 (3.2)		53.75 (15.6)	
>=51	20.19 (7.1)		24.75 (7.1)		13.38 (3.7)		58.31 (16.4)	
**Gender**								
Male	20.15 (5.5)	0.099	25.46 (5.9)	0.274	13.67 (4.7)	0.154	59.28 (14.7)	0.123
Female	18.57 (6.1)		24.39 (6.1)		12.84 (3.5)		55.80 (14.1)	
**Marital status**								
Single	18.93 (5.9)	0.306	24.88 (5.9)	0.553	12.84 (3.5)	0.773	56.65 (13.6)	0.52
Married	18.50 (6.2)		24.22 (6.4)		12.99 (3.9)		55.71 (14.9)	
Divorced/Widow	20.93 (5.4)		25.33 (4.2)		13.53 (2.1)		59.80 (10.0)	
**Nationality**								
Saudi	18.22 (5.1)	0.001	26.00 (6.0)	0.094	14.20 (3.3)	0.005	58.42 (12.6)	0.198
Indian	17.13 (5.6)		23.24 (6.4)		13.70 (3.9)		54.06 (15.0)	
Filipino	19.67 (6.3)		24.73 (6.1)		12.44 (3.6)		56.83 (14.3)	
Other	13.50 (4.2)		23.83 (2.8)		12.00 (3.3)		49.33 (9.3)	
**Level of nursing**								
Diploma	19.89 (6.9)	0.675	26.11 (3.7)	0.305	13.67 (3.2)	0.164	59.66 (11.7)	0.316
Bachelor	18.71 (6.0)		24.41 (6.1)		12.87 (3.5)		55.98 (14.0)	
Master	20.00 (6.7)		26.91 (8.2)		14.91 (6.6)		61.81 (20.7)	
**Nursing experience (in years)**								
< 1	19.46 (4.8)	0.432	25.17 (4.9)	0.247	13.37 (3.7)	0.395	58.00 (11.3)	0.279
5-Jan	19.23 (5.9)		25.32 (6.2)		13.33 (3.6)		57.89 (14.1)	
10-Jun	18.19 (6.4)		23.79 (6.5)		12.81 (3.9)		54.79 (15.3)	
15-Nov	17.95 (6.5)		23.55 (6.0)		12.25 (3.3)		53.74 (14.2)	
> 15	19.68 (6.1)		25.00 (6.6)		12.64 (3.9)		57.32 (14.7)	
**Working unit**								
Intensive Care unit	17.14 (5.5)	0.034	23.34 (5.6)	0.018	12.18 (2.8)	0.03	52.65 (12.5)	0.016
Cardiac intensive care unit	18.97 (5.4)		24.86 (6.1)		13.81 (4.5)		57.64 (14.5)	
Pediatric cardiac Intensive care unit	17.71 (4.6)		23.79 (4.3)		10.86 (2.7)		52.35 (9.3)	
Pediatric Intensive care unit	18.27 (4.7)		26.09 (3.4)		13.18 (3.1)		57.54 (8.9)	
Neonatal Intensive care unit	20.36 (6.5)		24.98 (6.0)		12.89 (3.3)		58.22 (14.1)	
Emergency room	19.12 (6.3)		25.33 (7.0)		13.71 (4.7)		58.16 (16.6)	
Medical ward	18.17 (6.5)		23.24 (5.8)		12.81 (3.4)		54.22 (14.3)	
Nephro ward	20.17 (5.9)		24.00 (5.6)		13.00 (2.5)		57.16 (12.0)	
Surgical ward	18.27 (5.9)		24.40 (6.6)		12.69 (3.5)		55.35 (14.2)	
VIP ward	15.22 (6.1)		20.89 (7.6)		10.33 (1.3)		46.44 (14.5)	
Pediatric ward	22.06 (6.2)		29.76 (4.4)		15.00 (4.0)		66.82 (12.7)	
Other	27.50 (3.5)		28.00 (0.0)		14.50 (0.71)		70.00 (4.2)	
**How many patients**								
1 to 5	18.89 (6.1)	0.629	24.65 (6.4)	0.9	13.00 (3.8)	0.677	56.52 (14.6)	0.734
6 to 10	18.98 (6.0)		24.43 (5.8)		13.10 (3.7)		56.50 (13.8)	
> 10	18.02 (5.9)		24.24 (5.7)		12.54 (3.3)		54.80 (13.3)	
**Have received training**								
Yes	18.23 (5.9)	0.354	24.23 (6.4)	0.614	13.54 (4.0)	0.099	56.01 (15.0)	0.853
No	18.95 (6.1)		24.63 (6.1)		12.77 (3.6)		56.35 (14.0)	
**Have received support**								
Yes	19.59 (6.4)	0.221	25.28 (6.0)	0.264	13.38 (3.6)	0.284	58.25 (14.2)	0.201
No	18.58 (5.9)		24.35 (6.2)		12.85 (3.7)		55.77 (14.2)	
**Type of interventions**								
Bereavement Counseling	20.09 (6.8)	0.449	25.11 (6.8)	0.835	12.51 (4.2)	0.518	57.71 (16.2)	0.82
One-to-One Debriefs with Team Leader or Other Leadership	20.07 (5.5)		25.73 (5.4)		13.20 (4.0)		59.00 (13.5)	
Psychological Therapy	18.22 (5.6)		24.26 (5.7)		13.44 (3.3)		55.92 (12.9)	
Spiritual Support	18.24 (6.5)		24.24 (7.2)		13.02 (4.1)		55.49 (16.5)	
Team Debriefs	18.65 (5.9)		24.32 (5.7)		12.55 (3.3)		55.52 (13.2)	
Other	18.86 (5.9)		25.29 (6.8)		14.00 (4.6)		58.14 (16.3)	

The relationships between the levels of grief trait, grief state, and total grief and their coping factors and total score mean values showed a highly statistically significant difference. The mean values of the three factors (problem-focused coping, emotion-focused coping, and avoidant coping) and the total coping score were significantly higher (*p* < 0.001) among nurses who had a high or severe level of grief trait, grief state, and total grief when compared to nurses with normal level of grief trait, grief state, and total grief (Table 6).

**Table 6:** Relationship between levels of grief trait, grief state and total grief and their coping factors and total scores

Grief and its components	Problem Focused Coping		Emotion Focused Coping		Avoidant Coping		Coping total score	
	Mean (Sd.)	*p*-value	Mean (Sd.)	*p*-value	Mean (Sd.)	*p*-value	Mean (Sd.)	*p*-value
**Grief Trait**								
Normal level	18.33 (6.1)	0.001	23.92 (5.9)	< 0.0001	12.42 (3.3)	< 0.0001	54.67 (13.8)	< 0.0001
High level	22.17 (5.2)		28.03 (5.6)		15.69 (3.8)		65.88 (12.5)	
Severe level	19.67 (4.9)		28.40 (6.7)		16.93 (5.0)		65.0 (15.8)	
**Grief State**								
Normal level	18.29 (6.1)	0.001	23.86 (5.9)	< 0.0001	12.44 (3.4)	< 0.0001	54.58 (13.8)	< 0.0001
High level	21.54 (5.2)		28.14 (5.3)		15.83 (3.3)		65.51 (11.2)	
Severe level	22.14 (5.6)		29.71 (6.9)		16.57 (5.2)		68.43 (16.4)	
**Total Grief**								
Normal level	18.30 (6.1)	0.001	23.87 (5.9)	< 0.0001	12.38 (3.3)	< 0.0001	54.55 (13.7)	< 0.0001
High level	22.0 (4.5)		28.10 (5.6)		16.22 (3.8)		66.31 (11.5)	
Severe level	21.25 (5.9)		29.69 (6.8)		16.88 (5.1)		67.81 (16.3)	

Correlation analysis showed a positive statistically significant linear correlation between the scores of the three coping factors (problem-focused, emotion-focused, and avoidant coping) and the total coping scores and grief trait, grief state, and total grief scores. That is, as the scores on the three factors and nurses’ total coping scores increased, their grief, grief, and total grief scores also increased, and all the correlation coefficients were statistically significant (Table 7).

**Table 7:** Correlation between three factors and total scores of nurses coping and their grief scores

*Coping* *Factors*	Grief trait score		Grief State score		Grief total score	
	*r*- value	*p*-value	*r*- value	*p*-value	*r*- value	*p*-value
Problem Focused Coping	0.257	< 0.0001	0.339	< 0.0001	0.331	< 0.0001
Emotion Focused Coping	0.352	< 0.0001	0.433	< 0.0001	0.432	< 0.0001
Avoidant Coping	0.479	< 0.0001	0.486	< 0.0001	0.521	< 0.0001
Coping total score	0.385	< 0.0001	0.456	< 0.0001	0.462	< 0.0001

The regression analysis revealed that 21.3% of the changes in total coping scores among nurses were explained by the values of the total grief score, which was statistically significant (F = 92.18, *p* < 0.0001) with R^2^ = 0.213. The regression coefficient of the total grief score (0.411, t = 9.601, *p* < 0.0001) explained that for every unit change in the total grief score, the total average coping score increased by 0.411 units.

## Discussion

This study assessed the relationship between grief and coping strategies among nurses dealing with patient deaths, revealing that nurses experiencing higher levels of grief tend to employ more coping strategies, suggesting a complex interplay between these two factors in the context of patient care, which is consistent with a systematic review of qualitative studies conducted by Zheng et al. [26], highlighting the challenges nurses face in coping with patient deaths and identifying similar areas that should be addressed to better help nurses cope with patient loss [26]. Our study adds nuances by examining specific coping strategies employed by nurses and their associations with grief levels.

We found significant associations among socio-demographic factors, professional characteristics, and grief levels. Socio-demographics included nationality (Saudis compared to expatriate nurses), received support, intervention type (psychological therapy), and place of employment (nurses working in pediatric units). In terms of nationality, Saudi nurses were at a higher risk for grief than nurses of other nationalities. Similar findings were reported by Sharif et al. [25], who examined the relationship between psychological symptoms and job satisfaction among nurses in Saudi Arabia, which showed higher levels of anxiety and depression among Saudi nurses. This finding could be explained by the socio-cultural diversity of death and dying and suggests a possible correlation between caring for people of the same nationality and increased grief level, which has not been fully explored in the literature and requires further study. Our sample was not sufficient to explore the relationship between grief and acceptance levels among male and female nurses or across different age groups. However, in other studies, older and male nurses showed less grief and higher acceptance of patient deaths than their female and younger peers [9, 10].

Received support was of particular significance in this study, where the risk of grief was significantly higher among nurses who did not receive support compared to those who did. This finding is consistent with those of Chang [27], who highlighted the positive impact of social support from peers and supervisors on nurses providing care to terminally ill patients and experiencing patients’ deaths. Nurses’ grief risk was further impacted by the department in which they worked. We found that nurses working in pediatric departments had a higher grief risk compared to those in other departments, which could be attributed to the strong emotions triggered by observing a patient die at a young age, as echoed in many studies in the literature [28, 29]. However, a study conducted by Das et al. [30] suggested that emotional connections with patients influence grief, irrespective of setting.

The linear relationship between grief and coping identified in this study suggests that nurses employ more coping strategies as their grief levels increase. This finding contrasts with the literature, indicating that increased grief leads to burnout and reduced productivity [31]. Our findings may be attributed to the role of religion, which emerged as the most commonly used coping strategy, providing comfort and meaning during times of loss. Many nurses found solace and strength in their faith through prayers, religious rituals, and faith-based support systems. The reliance on religious coping mechanisms is consistent with the findings of Ross et al. [32], who noted that healthcare professionals frequently draw on spiritual beliefs to manage stress and grief. The spiritual dimension provides a framework for understanding and processing loss, offering a sense of purpose and hope in challenging circumstances. Integrating spiritual support into workplace interventions could enhance nurses’ emotional resilience, particularly in culturally diverse settings where religion plays a pivotal role in daily life.

Cultural background, societal norms, and personal beliefs about death and dying influence emotional responses and coping mechanisms. Studies have shown that culturally tailored interventions can significantly enhance coping strategies and mental health outcomes for healthcare professionals [33, 34]. In cultures that emphasize community support and collective grieving, nurses may find it easier to share their feelings and receive emotional support. The relevance of these interventions is particularly relevant in Saudi Arabia, where cultural and religious beliefs heavily influence perceptions of death and the grieving process [35].

Based on our findings, we recommend the development of evidence-based interventions tailored to the unique needs of nurses dealing with patient death. These interventions should operate at both the individual and organizational levels, focusing on fostering resilience and promoting mental health awareness in healthcare settings. However, further exploration is needed, specifically identifying effective modalities of support, such as counseling, peer support groups, and stress management training. Furthermore, consideration is needed regarding the timing, cultural accessibility, and different experiences across different hospital wards or units.

### Strengths and limitations

The study benefits from being adequately powered within a robust research design and a diverse sample. However, we did not capture any open-text responses that may have provided beneficial contextual nuances and insights which represents an important consideration for future studies. Additionally, the study’s focus on a single medical center may limit the generalizability of the findings, and future research should include multisite and multicultural exploration to provide a more comprehensive understanding.

## Conclusions

This study explored the complex relationship between grief and coping in the context of patient death among nurses at a Saudi Arabian hospital. Our findings highlight the need to identify factors influencing nurses’ experiences of grief and coping and to provide support systems for nurses dealing with patient death, enhancing nurses’ well-being and improving patient care outcomes. Further research is needed to tailor interventions effectively, ensuring that nurses are better supported in managing their grief and promoting their overall well-being in the workplace.

## Data Availability

The data that support the findings of this study are available from the corresponding author [LS] upon reasonable request.

## Abbreviations

Brief-COPE: Brief Coping Orientation to Problems Experienced Inventory
GSSN: Grief Traits and State Scale for Nurses
VIP: Very important person
